# Parthenolide induces rapid thiol oxidation that leads to ferroptosis in hepatocellular carcinoma cells

**DOI:** 10.3389/ftox.2022.936149

**Published:** 2022-12-14

**Authors:** Francesca V. LoBianco, Kimberly J. Krager, Erica Johnson, Christopher O. Godwin, Antino R. Allen, Peter A. Crooks, Cesar M. Compadre, Michael J. Borrelli, Nukhet Aykin-Burns

**Affiliations:** ^1^ Division of Radiation Health, Department of Pharmaceutical Sciences, University of Arkansas for Medical Sciences, Little Rock, AR, United States; ^2^ Department of Pharmaceutical Sciences, University of Arkansas for Medical Sciences, Little Rock, AR, United States; ^3^ Department of Radiology, University of Arkansas for Medical Sciences, Little Rock, AR, United States

**Keywords:** hepatocellular carcinoma (HCC), parthenolide (PTL), thiol oxidation, lipid peroxidation, ferroptosis, glutathione

## Abstract

Hepatocellular carcinoma (HCC) is both a devastating and common disease. Every year in the United States, about 24,500 men and 10,000 women are diagnosed with HCC, and more than half of those diagnosed patients die from this disease. Thus far, conventional therapeutics have not been successful for patients with HCC due to various underlying comorbidities. Poor survival rate and high incidence of recurrence after therapy indicate that the differences between the redox environments of normal surrounding liver and HCC are valuable targets to improve treatment efficacy. Parthenolide (PTL) is a naturally found therapeutic with anti-cancer and anti-inflammatory properties. PTL can alter HCC’s antioxidant environment through thiol modifications leaving tumor cells sensitive to elevated reactive oxygen species (ROS). Investigating the link between altered thiol mechanism and increased sensitivity to iron-mediated lipid peroxidation will allow for improved treatment of HCC. HepG2 (human) and McARH7777 (rat) HCC cells treated with PTL with increasing concentrations decrease cell viability and clonogenic efficiency *in vitro*. PTL increases glutathione (GSH) oxidation rescued by the addition of a GSH precursor, N-acetylcysteine (NAC). In addition, this elevation in thiol oxidation results in an overall increase in mitochondrial dysfunction. To elucidate if cell death is through lipid peroxidation, using a lipid peroxidation sensor indicated PTL increases lipid oxidation levels after 6 h. Additionally, western blotting reveals glutathione peroxidase 4 (GPx4) protein levels decrease after treatment with PTL suggesting cells are incapable of preventing lipid peroxidation after exposure to PTL. An elevation in lipid peroxidation will lead to a form of cell death known as ferroptosis. To further establish ferroptosis as a critical mechanism of death for HCC *in vitro*, the addition of ferrostatin-1 combined with PTL demonstrates a partial recovery in a colony survival assay. This study reveals that PTL can induce tumor cell death through elevations in intracellular oxidation, leaving cells sensitive to ferroptosis.

## 1 Introduction

Hepatocellular carcinoma (HCC) is the cancer of primary liver cells and is estimated to result in <1 million new cases by 2025 (Llovet et al., 2021). There are various risk factors associated with HCC, including cirrhosis, viral hepatitis, alcohol, obesity, and nonalcoholic fatty liver disease (NAFLD) (Huang et al., 2021). An overarching mechanism associated with these risk factors is an injury to the liver’s normal parenchyma that results in progressive inflammation and regeneration, leading to the eventual development of HCC (Marrero, 2020). The 5-year relative survival for patients diagnosed with HCC is only 18% and worse for patients with less localized tumors (Siegel et al., 2019). A contributing factor to this poor survival is that HCC is typically diagnosed late in its course within the setting of chronic liver disease. The typical late-stage diagnosis in HCCs leads to a median survival following diagnosis of 6–12 months (Investigators, 1998). While there are various treatment options for HCC, not all options are available to patients due to the severity of HCC and the amount of healthy liver present (El-Serag et al., 2008). HCC is a drug-resistant tumor due to the high expression of multi-drug resistant proteins and elevated reactive oxygen species (ROS) (Ng et al., 2000; Ogunwobi et al., 2019). Overall, there is a need for new therapeutics that can be affordable, effective, and delivered locoregionally for HCC.

Parthenolide (PTL) is a naturally occurring sesquiterpene lactone derived from the plant known as feverfew (*Tanacetum parthenium*). PTL has been used to treat arthritis, insect bites, infections, headaches, and colds (Chavez and Chavez, 1999; Jain and Kulkarni, 1999). However, more recently, PTL was found to have anti-cancer and pro-apoptotic abilities (Wen et al., 2002). Previous studies attribute PTL’s cytotoxic activity to the reaction of the lactone moiety with thiols, such as proteins that contain cysteine residues or sulfhydryl groups (-SH) (Herz, 1977; Zhang et al., 2004). Glutathione (GSH) is a vital thiol and redox substrate essential for the detoxification of ROS within the cell and is controlled by the rate of oxidation, extrusion, uptake of thiol precursors, and synthesis (Fujii et al., 2011). In colorectal cancer cells, depletion of thiol groups on the mitochondrial membrane damages the mitochondrial membrane’s integrity causing a vicious cycle of oxidative stress-induced cancer cell death (Zhang et al., 2004). Previous research demonstrated that PTL activated NADPH oxidases, increased ROS within prostate cancer cells, and suppressed antioxidants to generate elevated oxidative stress (Sun et al., 2010). In this study, we demonstrate that not only does PTL result in a decrease in the level of GSH and elevation of ROS within cancer cells, but this depletion of GSH results in a downstream form of cell death known as ferroptosis.

Ferroptosis is a form of regulated cell death defined as a result of missing or insufficient activity of the selenoperoxidase, glutathione peroxidase 4 (GPx4), leading to increased levels of ROS and cell death through lipid membrane oxidation (Yu et al., 2016; Lei et al., 2019; Mou et al., 2019). Ferroptosis is prevented by cells importing cysteine through the Xc-transport system to synthesize glutathione for GPx4 activity. Extracellular cysteine is imported *via* a cysteine/glutamate antiporter. The cysteine is then utilized to form GSH, the substrate for GPx4. GPx4 converts PUFA-OOH to PUFA-OH reducing the number of possible ROS in the cell. PTL reacts with GSH preventing adequate GPx4 function and leading to oxidative damage and eventual cell death through ferroptosis. Current drugs used to treat cancer through ferroptosis include erastin, sorafenib and sulfasalazine which inhibit the Xc-system by inhibiting cysteine transport thereby blocking GSH synthesis and GPx4 function (Yu et al., 2016; Mou et al., 2019).

GSH is the essential substrate for GPx4, where lowering GSH intracellularly results in a form of death morphologically identical to the death induced by loss of GPx4 (Dixon et al., 2012). Typically in normal cells, antioxidant mechanisms efficiently prevent lipid peroxidation initiation by GPx4 activity, which uses GSH (Scott, 1997). Interestingly, while cancer typically has high levels of ROS and dysregulated redox metabolism, ferroptosis does not frequently occur during tumorigenesis. Furthermore, triggering ferroptosis induces death in therapy-resistant cancer due to cancer cells relying on GPx4 for survival (Hangauer et al., 2017). A reason for this GPx4 dependency is that cancer cells rely on iron at a greater level than normal cells for growth, metabolism, and metastasis resulting in iron being available as a catalyst for rapid lipid peroxidation if the lipid membrane is not protected by GPx4 (Liu et al., 2018). Recently, PTL was linked to ferroptosis in triple-negative breast cancer cells (TNBC) through ROS elevation and ubiquitination of GPx4 (Ding et al., 2021). However, no studies focus on ferroptosis as a mechanism of death from sesquiterpene lactones in HCC. This study reveals that PTL induces ferroptosis in HCC following GSH depletion, rapid oxidation of intracellular and mitochondrial thiols, mitochondrial dysfunction, and suppression of GPx4.

## 2 Materials and method

### 2.1 Cell and culture conditions

Cells selected for *in vitro* experiments included human hepatocellular carcinoma (HepG2) and rat Morris hepatoma (McA-RH7777) cells were obtained from the American Type Culture Collection (ATCC, Manassas, VA, United States). Cells were cultured in their respective media Minimum Essential Media (MEM) and Dulbecco’s Modified Eagle Medium (DMEM) supplemented with 10% fetal bovine serum (FBS) and 100 U/ml penicillin and 100 μg/ml streptomycin (P/S). Cells were maintained in a 5% CO_2_ humidified atmosphere at 37°C. Cells were collected for experiments using 0.25% trypsin and counted using trypan blue stain with the Countess Cell Counter to confirm 90% or better viability. PTL (Sigma, Burlington, MA, United States) was obtained and solubilized in dimethylsulfoxide (DMSO) to 25 mM concentration and then diluted to working concentrations in PBS. The final concentration of vehicle DMSO treatment groups is 0.05%.

### 2.2 Isolation of murine primary hepatocytes

Primary hepatocytes were isolated from C57BL/6 mice by cannulation through the inferior vena cava to perfuse the liver using oxygenated calcium free perfusion buffer at 37°C (Hanks Balanced Salt Solution, 5.8 mM HEPES, 4.5 mM NaHCO_3_). After the liver tissue was perfused, oxygenated digestion buffer (perfusion buffer with 5 mM CaCl_2_, 15 mg collagenase, and 25 mg trypsin inhibitor) at 37°C was added until the liver began to digest. The liver was further digested by removing the liver and placing it in a petri dish with un-supplemented F-12 media. Cells were then spun at 4°C for 3 min at 800 rpm twice. Cells were resuspended in 25 ml un-supplemented F-12 media and 25 ml of 90% PerColl and centrifuged at 4°C for 8 min at 140 g to remove debris and dead cells. Cells were then resuspended in supplemented (10% FBS, 100 U/ml penicillin and 100 μg/ml streptomycin) dye free RPMI 1640 media and centrifuged for 3 min at 800 rpm to remove residual PerColl. The pellet was resuspended in complete RPMI 1640 media and counted with trypan blue staining. Only collections of 90% viability or above were used for experiments. After collection 10,000 cells per well were plated on a collagen IV coated 96 well plate for further experiments.

### 2.3 MTT assay

3-(4,5-dimethylthiazol-2-yl)-2,5-diphenyltetrazolium bromide (MTT) assay was used to evaluate the dose of PTL required for further cell culture experiments. HepG2, McARH-7777, and isolated primary hepatocytes were plated on a 96 well plate at 3,000–10,000 cells per well. Treatment groups were completed in quadruplicates with increasing amounts of PTL along with vehicle control (0.05% DMSO), media control, and toxic 10% DMSO. Plates were exposed to treatment for 24 h. An additional plate of HepG2 cells was treated with 10 mM NAC 24 h prior to treatment with PTL. Cells were incubated with MTT (500 μg/ml final concentration in the wells) at 37°C for 4 h. The MTT containing media was removed, DMSO was added into the wells and plates were placed on an orbital shaker for 5 min before absorbance was measured at 490 nm using BioTek plate reader. The MTT reduction (% of control) was calculated for each group and placed on a logarithmic scale to determine the IC50 dose and IC90 dose for each cell line by nonlinear regression analysis for future experiments.

### 2.4 Clonogenic survival assay

HepG2 and McARH-7777 cells were treated with 500 nM of ferrostatin-1 24 h prior to treatment with PTL at IC50 and IC90 for 3 h and 6 h. After treatment cells were trypsinized, re-plated at different clonogenic densities, and left for 10–14 days in a 37°C incubator. The colonies were fixed with 70% ethanol and stained with Coomassie brilliant blue stain (prepared in 50% methanol, 5% acetic acid, 45% H_2_O) and counted using a microscope. Colonies were defined as 50 cells per colony and percent survival was determined.

### 2.5 Thiol oxidation status of cells

HepG2 and McARH-7777 were treated with 50–100 MOI of either cytosolic or mitochondrial roGFP adenovirus following previously published protocol (Aliyev et al., 2020). Cells were then treated with IC50 and IC90 doses of PTL. Alterations of fluorescence wavelength frequencies between oxidized and reduced were measured and quantified using fluorescent microscopy for visualization and representative imaging. Quantification of the cells after time points 30 min, 1 h, 2 h, 4 h, 6 h, and 24 h was determined by flow cytometry (BD LSRFortessa) gated channels excitation 405 nm/emission 525 nm (Brilliant Violet 510 [BV510]) and excitation 488 nm/emission 525 nm (fluorescein isothiocyanate [FITC] with assistance from the UAMS Molecular Imaging Core Facility. Each cell’s ratio of fluorescence from excitation at 405 and 488 nm, which cancels out the amount of indicator and the absolute optical sensitivity, shows how much oxidation is occurring.

### 2.6 Lipid peroxidation assessment

HepG2 cells were treated at IC50 and IC90 of PTL at 30-min, 3 h, and 6 h time points. 10 µM of the Image-iT Lipid Peroxidation Sensor (Invitrogen, Waltham, MA, United States) was added to the cells and incubated for 30 min, cells were washed with PBS. The ratio of oxidized and reduced was determined by the excitation 488 nm/emission 510 nm (TexasRed) and excitation 581 nm/emission 591 nm (FITC) by flow cytometry (BD LSRFortessa) assistance from the UAMS Molecular Imaging Core Facility.

### 2.7 Total and oxidized glutathione measurement

1 × 10^6^ HepG2 cells were treated with PTL in triplicate at the IC50 and IC90 dose for 3 h, 6 h, and 24 h on two separate days. An additional group of cells was treated with 10 mM NAC 24 h prior to treatment with PTL in triplicate for the same time points above. Cells were collected and diluted in 1.34 mM DETAPAC (diethylenetriaminepenta-acetic acid) buffer on ice. Cells were lysed in 5% sulfosalicylic acid and centrifuged. The 5,5′-dithiobis-2-nitrobenzoic acid recycling assay was performed to measure total GSH and GSSG levels (nmol/mg protein) using a Perkin Elmer UV/Vis Spectrophotometer (Griffith, 1980). Data were normalized to protein content determined by Lowry’s protein assay (Lowry et al., 1951).

### 2.8 Measurement of mitochondrial function

HepG2 cells were plated 10,000 per well on a Celltak coated plate and media was changed to unbuffered DMEM containing 4 mM glutamate and incubated in a non-CO_2_ incubator at 37°C for 1 h. IC50 and IC90 dose was injected into each well and four baseline measurements were acquired before injection of mitochondrial inhibitors or uncouplers. Readings were taken after the sequential addition of oligomycin (10 µM), FCCP (5 µM), and rotenone/antimycin A (10 µM), and measurements were taken over 24 h. The oxygen consumption rate (OCR) was measured at 37°C using an XF96 extracellular flux analyzer (Agilent Technologies). OCR was calculated by the Seahorse XF96 software and represents an average of 20–32 measurements on different days (Ferrick et al., 2008).

### 2.9 Western blotting

HepG2 cells were plated 1 × 10^6^ and treated with IC50 and IC90 doses for 1 h, 3 h, 6 h, and 24 h, and the cells were collected. The protein concentrations were analyzed using the Pierce BCA protein assay kit and standard curve (23225, Thermo Fisher, United States). Protein samples were then diluted in 1X Laemmli sample buffer with 5% 2-beta mercaptoethanol (2-BME) and evenly loaded with 25 µg of protein per lane in a 5–14% gradient Mini-PROTEAN TGX gel (456-1034 or 456-1084, Bio-Rad, United States). The gels were run for 60 min at 90 mV and transferred to nitrocellulose blot paper and run for 60 min at 100 mV at 4°C. The blots were blocked for 1 h in Odyssey Blocking Buffer (LI-COR, Lincoln, NE, United States) then incubated in a 1:1000 dilution of the target antibody GPx4 overnight (ab125066, Abcam, MA, United States). The loading control antibody lamin-A/C was incubated for 3 h (4777, Cell Signaling, MA, United States). After washing, the blots were incubated in a 1:10,000 dilution of LI-COR anti mouse/anti-rabbit fluorescent antibodies for 40 min. Blots were stored in 1X PBS. Imaging was visualized by using an Odyssey Fc Imaging System (LI-COR, Lincoln, NE, United States). Blots were exposed for 2 minutes each in the 700 and 800λ channels. Analysis of densitometry was completed with LI-COR software.

### 2.10 Statistical analysis

GraphPad Prism version 9.1.2 was used to perform the statistical analysis. Data are expressed as mean ± SEM. Statistical significance was determined at *p* < 0.05 and is expressed as *p < 0.05, **p < 0.01, ***p < 0.005. IC50 values were determined by non-linear regression analysis from plots of MTT reduction (% of control) versus LogC (M) drug concentration and an unpaired two-tailed *t*-test was performed between triplicate measures of IC50 for comparison of PTL and NAC. Significance was determined using a one-way ANOVA with Tukey’s post-analysis for thiol oxidation, GSH/GSSG measurements, lipid peroxidation, GPx4 protein levels, and oxygen consumption rate. A two-way ANOVA with Sidak’s multiple post-hoc comparison was used to analyze ferrostatin-1 rescue clonogenic assay to the PTL clonogenic assay.

## 3 Results

### 3.1 PTL is cytotoxic to HCC cells

**FIGURE 1:**
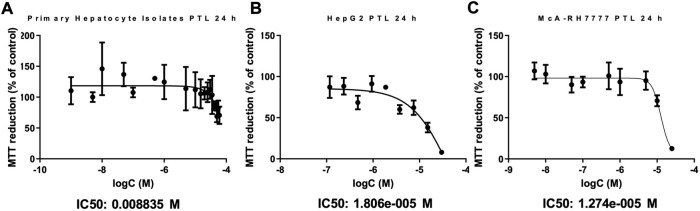
. PTL is substantially less toxic to primary murine hepatocytes **(A)** than to HepG2 **(B)** and McARH7777 **(C)** cells. MTT assay was used to estimate the cell viability in Primary murine hepatocytes **(A)**, HepG2 (human) **(B)**, and McARH7777 (rat) **(C)** cells. Plates were incubated with increasing concentrations of PTL in quadruplicate for 24 h in three different experiments (n=12). Nonlinear regression analysis was performed to determine IC50 and IC90 dose of PTL for each. **(A)** IC50: 8.8 mM, IC90: 26 mM. **(B)** IC50: 18 µM, IC90: 54 µM. **(C)** IC50: 13 µM, IC90: 40 µM.

HepG2 and McARH-7777 cells were exposed to increasing concentrations of PTL to determine appropriate concentrations for future experiments. DMSO that was used as the vehicle did not alter cytotoxicity or any other parameters we measured throughout the study (Supplemental Figures S1–S3). The MTT reduction (% of control) steadily decreases as the concentration of PTL increases. An IC90 dose was chosen to be three times as high as the IC50 for experiments to reveal differences in cellular response at 24 h. Figure 1B HepG2 IC50:18 μM, IC90: 54 µM and Figure 1C McA-RH7777 IC50:13 µM and IC90: 40 µM. Importantly, IC50 doses between human and rat HCC lines were similar at 18 μM and 13 µM respectively. Additionally, PTL exposed to the murine primary hepatocyte isolates suggested that PTL is less toxic to normal liver tissue than to the cancer cell lines as indicated by an IC50 dose of 8.8 mM (Figure 1A).

### 3.2 PTL rapidly oxidizes intracellular thiols

**FIGURE 2:**
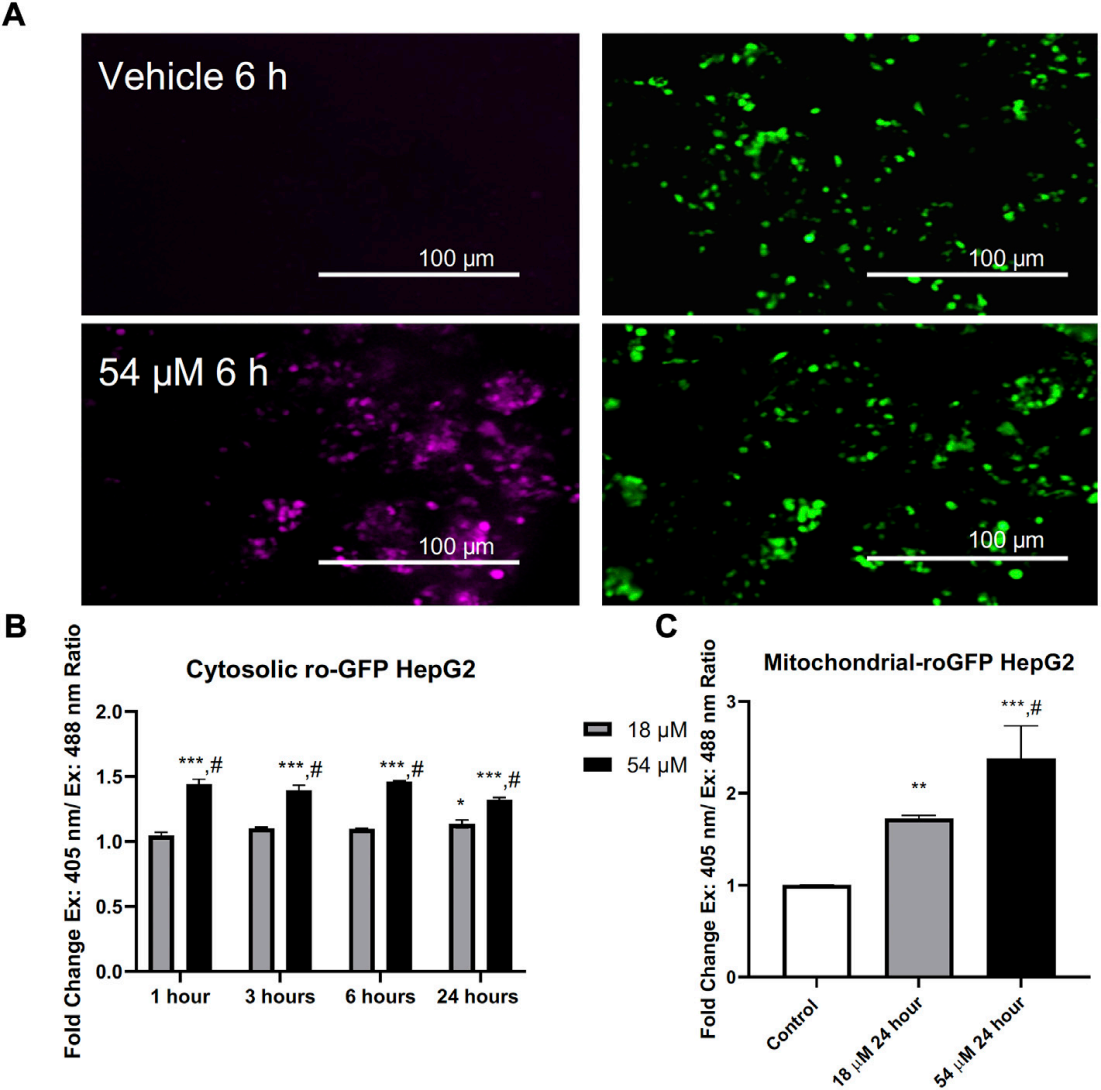
PTL induces an elevation of both cytosolic **(B)** and mitochondrial **(C)** thiol oxidation. **(A)** Representative imaging acquired by fluorescent microscopy. **(B)** Adenovirally expressed ratiometric oxidation status marker RoGFP measured with flow cytometry ex. 405 nm/em. 525 nm and ex. 488 nm/em. 525 nm reveals an elevation in the overall intracellular oxidation in response to PTL as early as 1 h posttreatment. **(C)** PTL resulted in an elevation of mitochondrial thiol oxidation after 24 h. (n=3). Errors represent ± 1 SEM. Significance determined by one-way ANOVA (# *p* < 0.05) with Tukey’s post-analysis comparing control to IC50 (18 µM) and IC90 (54 µM) * *p* < 0.05, ** *p* < 0.01, *** *p* < 0.001.

Thiol oxidation is considered to be an important mechanism for PTL’s rapid interaction within cells. RoGFP is a sensor that can be delivered to cells adenovirally to assess live cell thiol oxidation as it occurs (Aliyev et al., 2020). Figure 2 demonstrates PTL’s ability to rapidly oxidize cytosolic thiols within HepG2 cells, that is sustained over 24 h. Since PTL in previous research was found to result in mitochondrial alterations (Zhang et al., 2004), we used a mitochondrial roGFP sensor to assess mitochondrial thiol oxidation and found that at the IC90 dose there was an almost three-fold increase in oxidation of mitochondrial thiols after 24 h (Figure 2C). PTL is causing sustained oxidation to mitochondrial thiols that could lead to dysfunction of mitochondrial processes.

### 3.3 A major thiol, glutathione (GSH) is oxidized by PTL, and N-Acetylcysteine will decrease the amount of oxidation

**FIGURE 3:**
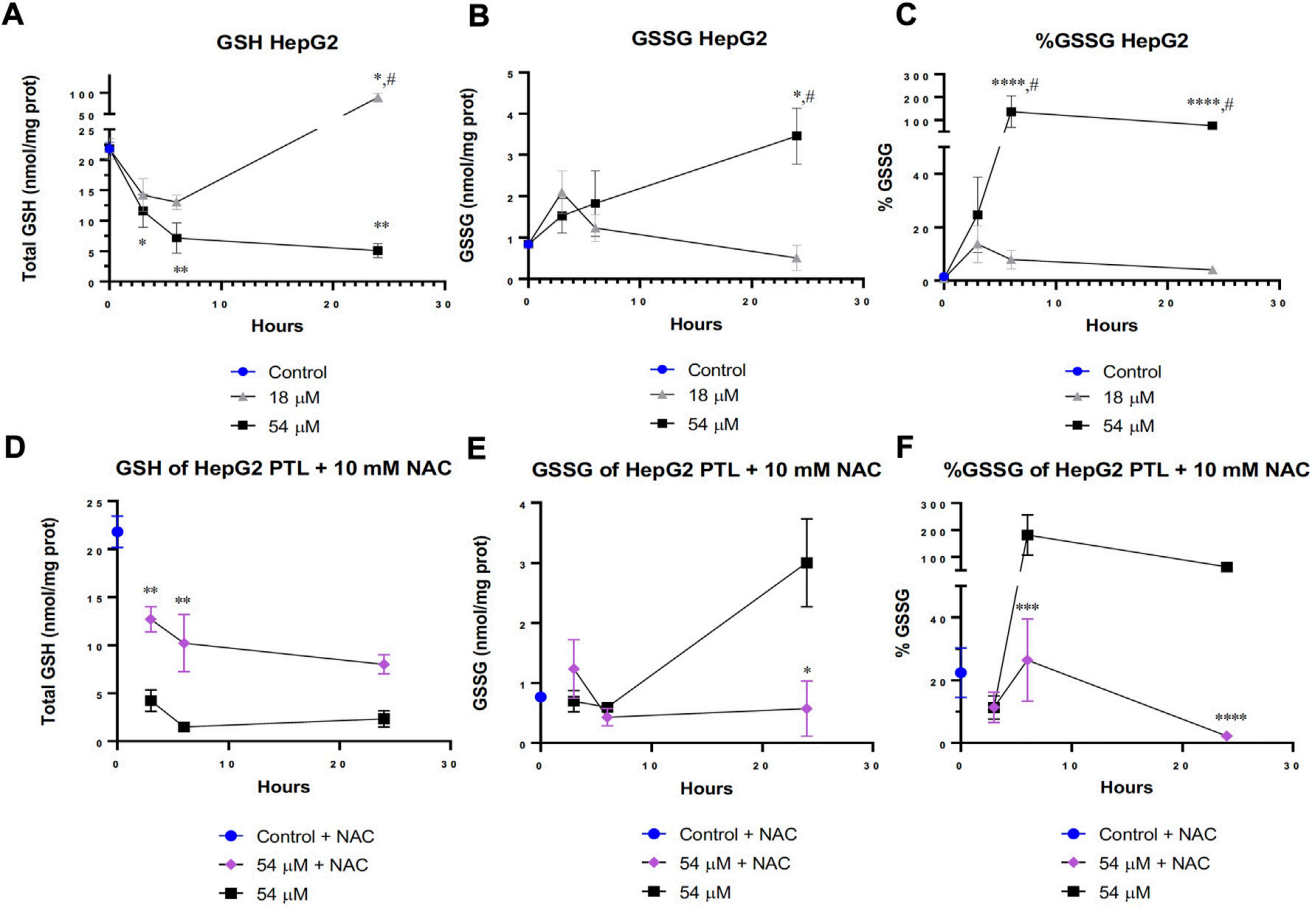
PTL increased glutathione (GSH) oxidation in HepG2 cells **(B,C)**. Cells attempted to overcome oxidation by elevating reduced GSH at the IC50 (18 µM) dose of PTL **(A)**. However, the IC90 (54 µM) dose prevented a compensatory response to increased oxidation and led to cell death **(A)**. 10 mM NAC rescued the level of GSH in cells treated with the IC90 dose **(D)** and decreased the level of GSH oxidation **(E,F)**. **(A–C)** HepG2 cells were treated with PTL (IC50 or IC90) in triplicate for each timepoint on 2 separate days (n=6). GSH **(A)**, GSSG **(B)** and % GSSG **(C)** normalized to protein content. Errors represent ± 1 SEM. Significance determined by one-way ANOVA (# *p* < 0.05) with Tukey’s post-analysis comparing control to IC50 (18 µM) and IC90 (54 µM) * *p* < 0.05, ** *p* < 0.01, *** *p* < 0.001. **(D–F)** HepG2 cells were treated with 10 mM NAC 24 h prior to treatment with IC90 dose of PTL in triplicate for each timepoint on 2 separate days (n=6). GSH **(A)**, GSSG **(B)** and % GSSG **(C)** normalized to protein content. Errors represent ± 1 SEM. Significance determined by one-way ANOVA with Tukey’s post-analysis between IC90 (54 µM) and NAC-IC90 * *p* < 0.05, ** *p* < 0.01, *** *p* < 0.001.

One major thiol within the cell is GSH. GSH plays an invaluable role in much of the cell’s basic redox homeostasis as it can be continually oxidized and reduced by enzymes within the cell to remove reactive oxygen species (ROS) (Forman et al., 2009). Interestingly, Figures 3A–C demonstrates a difference in the cellular response to PTL between the IC50 dose and IC90 dose. When cells were exposed to the IC50 dose for 24 h, the cells responded by elevating the level of reduced GSH (Figure 3A) and decreasing the amount of oxidized GSH also known as GSSG (Figures 3B,C). The cells were able to overcome rapid oxidation of GSH through compensation mechanisms however when the cells were treated with the IC90 dose over 24 h, the cells were unable to overcome this increased oxidation of GSH indicated by the decrease in GSH and elevation in GSSG.

When a glutathione precursor known as N-acetylcysteine (NAC) was added 24 h prior to PTL treatment, the cells were able to elevate their levels of GSH in response to the amount of oxidation occurring from PTL (Figure 3D). Additionally, Figures 3E,F displays less oxidation of GSH into GSSG after 24 h of PTL. Interestingly, the addition of NAC to cells treated with the IC90 dose behaved similarly to the IC50 treated cells in Figures 3A–C by having more GSH present within the cell. Figures 3D–F demonstrates that NAC was able to effectively salvage the levels of GSH and thiol oxidation but not completely prevent the oxidation present at the IC90 dose.

### 3.4 NAC rescues cells from PTL toxicity

**FIGURE 4:**
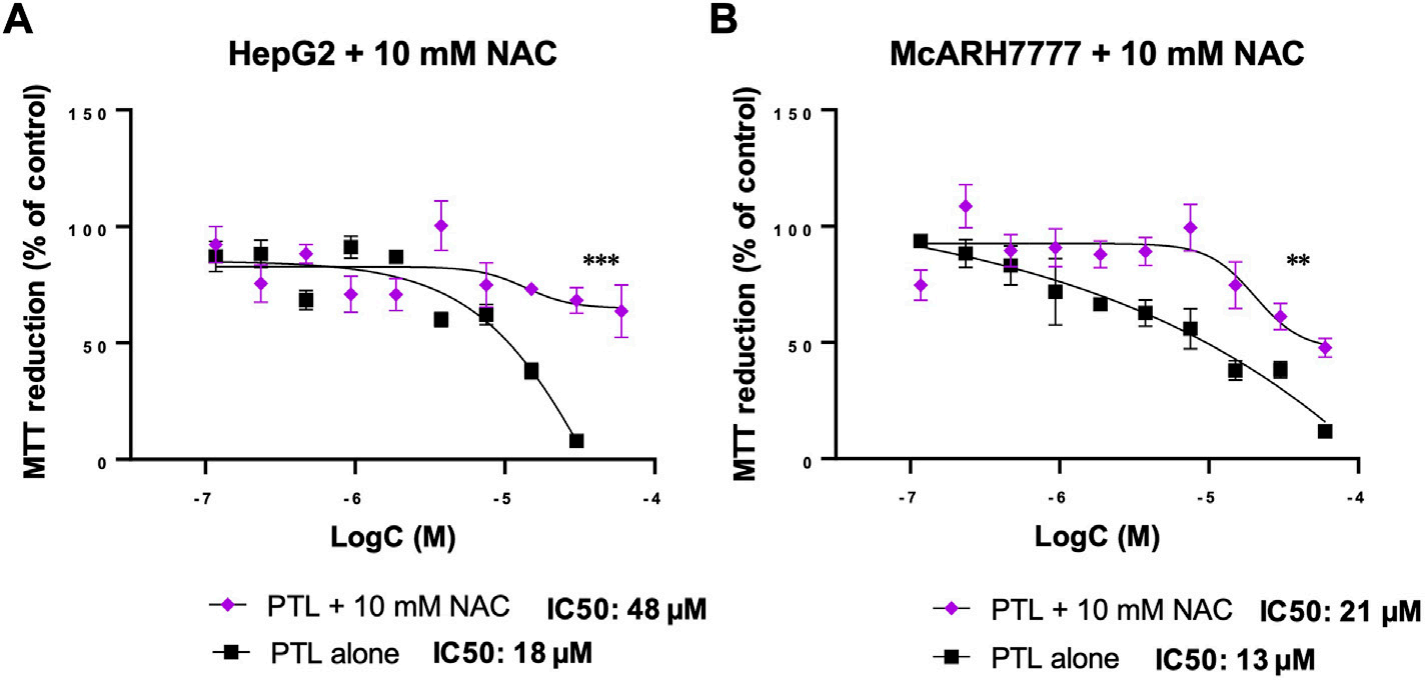
10 mM NAC reversed the decrease in MTT reduction (% of control) of HepG2 **(A)** and McARH7777 **(B)** cells with increasing concentrations of PTL. NAC increased the IC50 dose of PTL to 48 µM for HepG2 **(A)** and 21 µM for McA-RH7777. MTT assay was used to estimate the cell viability in HepG2 **(A)** and McARH7777 **(B)**. Plates were incubated with 10 mM NAC 24 h prior to treatment with increasing concentrations of PTL in quadruplicate for 24 h in 3 independent experiments. Nonlinear regression analysis was performed to determine the IC50 dose of PTL for each (n=12). Errors represent ± 1 SEM. Significance determined by unpaired two-tailed t-test between triplicate measures. **(A)** IC50 (18 µM) and NAC-IC50 (48 µM) for HepG2. **(B)** IC50 (13 µM) and NAC-IC50 (21 µM) for McA-RH7777 ** *p* < 0.01, *** *p* < 0.001.

As we demonstrated in Figure 3, NAC can protect HCC cells from GSH oxidation. Figure 4 demonstrates the prevention of thiol oxidation results in a protection from PTL cytotoxicity in both human (Figure 4A) and rat (Figure 4B) HCC cells. This addition of 10 mM NAC was able to increase the cell’s ability to reduce MTT even at the highest dose of PTL and significantly increases the IC50 dose required to reach the same toxicity. For HepG2 cells the IC50 dose for PTL is 18 µM but with the addition of NAC the IC50 dose is 48 µM (Figure 4A). For McA-RH7777 cells the IC50 dose for PTL is 13 µM but with the addition of NAC the IC50 dose increases to 21 µM (Figure 4B).

### 3.5 PTL increases lipid peroxidation and decreases the amount of GPx4 present in HCC cells

**FIGURE 5:**
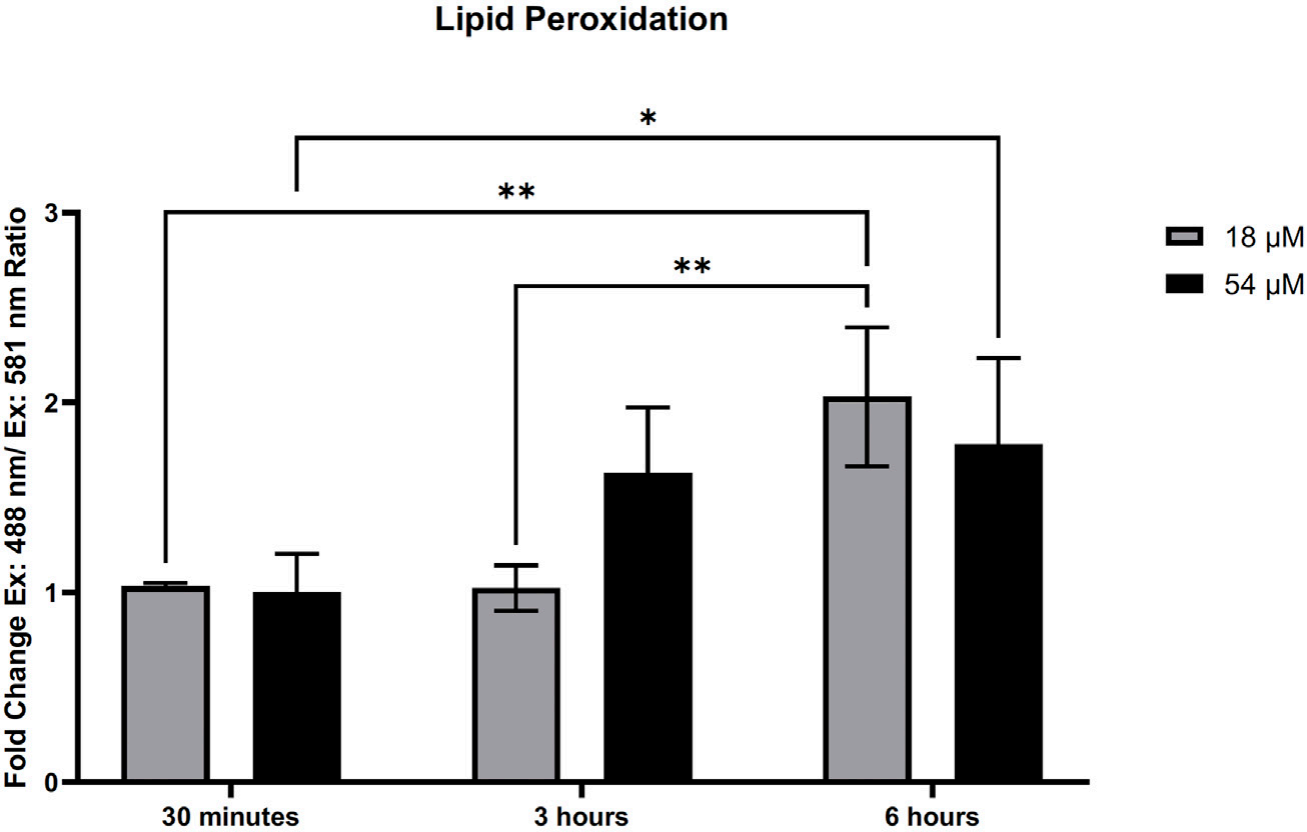
PTL induced lipid peroxidation in HepG2 cells. Lipid peroxidation measured by Image-IT sensor and flow cytometry demonstrated an elevation in lipid peroxidation after treatment with PTL. The ratio of oxidized and reduced was determined by the ex. 488 nm/em. 510 nm and ex. 581 nm/em. 591 nm. Fold-change was determined by comparison to the vehicle controls (n=3). Errors represent ± 1 SEM. Significance was determined by one-way ANOVA with Tukey’s post-analysis. IC50 (18 µM) and IC90 (54 µM) * *p* < 0.05, ** *p* < 0.01.

GSH plays an important role with GPx4 to remove lipid oxidation that may occur within the cell. Since we found rapid oxidation of thiols that resulted in a decrease of GSH within HepG2 cells, we assessed the amount of lipid peroxidation present. Figure 5 demonstrates that lipid peroxidation increased after 6 h of exposure to both IC50 and IC90 doses in HepG2 cells. Elevated lipid peroxidation is a notable mechanism that initiates the form of cell death known as ferroptosis. Similar to what was recently discovered in triple-negative breast cancer cells (Ding et al., 2021), Figure 6 demonstrates that in HepG2 cells, GPx4 protein levels were significantly decreased but only at the IC90 dose after 24 h. Interestingly, the IC50 dose allowed for recovery of the GPx4 levels which may account for some of the differences in response between the two doses seen.

**FIGURE 6:**
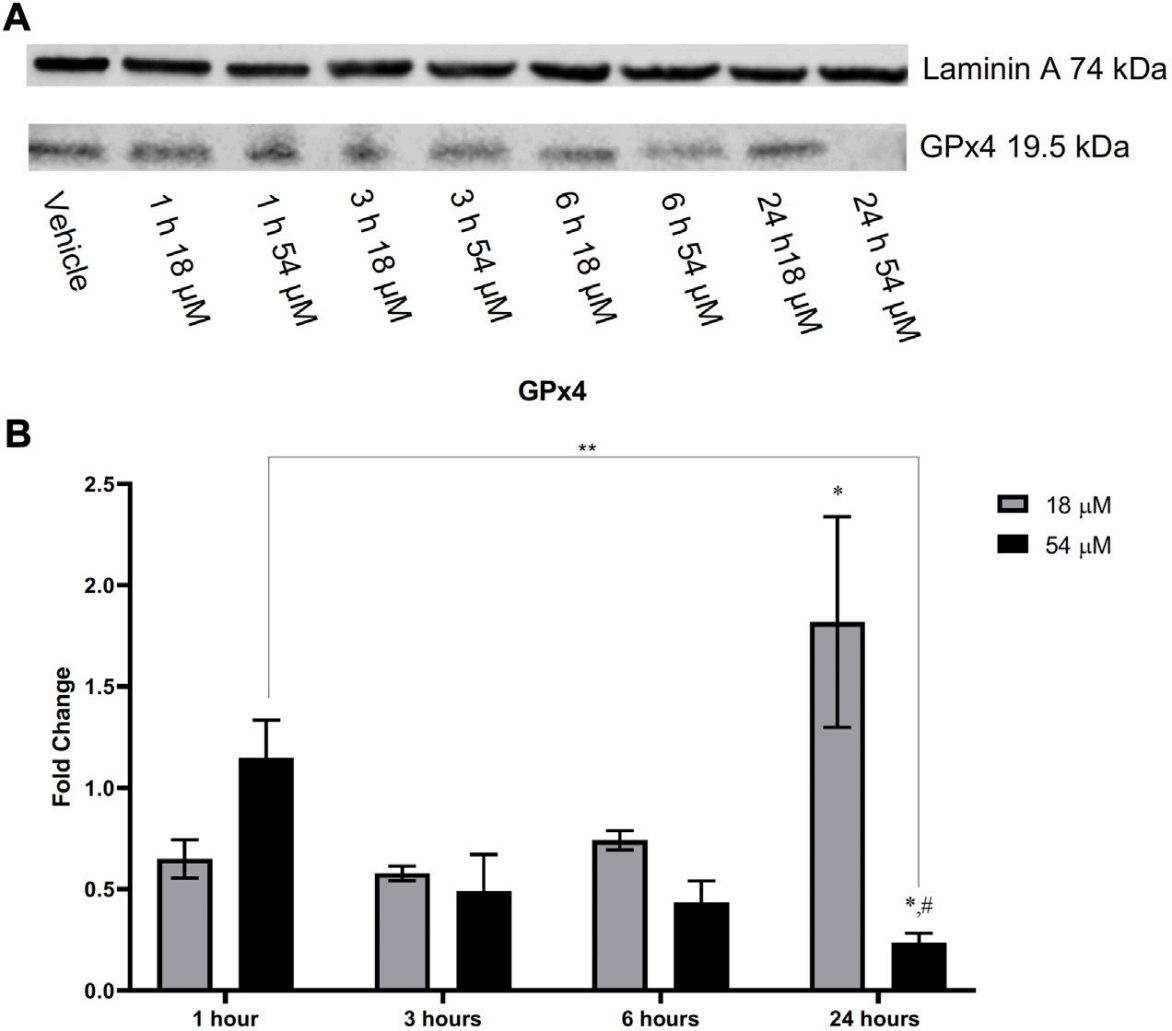
HepG2 cells after treatment with PTL demonstrated a decrease in GPx4 when exposed to the IC90 (54 µM) dose of PTL. Cells exposed to the IC50 (18 µM) dose appear to increase the level of GPx4 present over time. Imaging **(A)** and analysis **(B)** were obtained using Li-Cor imaging software. Blot was normalized to loading control. Fold-change was determined by comparison of densitometry to the vehicle controls (n=3). Errors represent ± 1 SEM. Significance determined by one-way ANOVA (# p < 0.0001) with Tukey’s post analysis comparing control to IC50 (18 µM) and IC90 (54 µM) * *p* < 0.05. Significant difference between IC90 1 h and IC90 24 h ** *p* < 0.01. # *p* < 0.0001 represents a significant difference between IC50 and IC90.

### 3.6 Inhibition of ferroptosis partially protects cells from PTL

**FIGURE 7:**
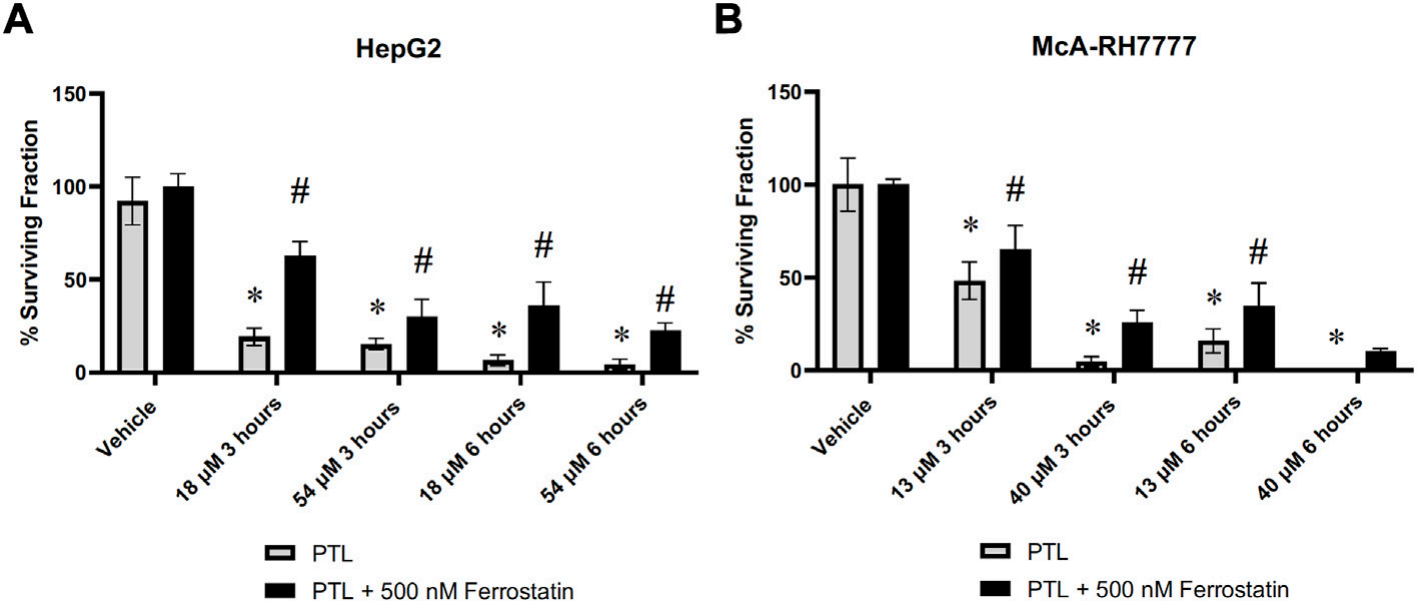
HepG2 **(A)** and McA-RH7777 **(B)** cells were rescued by ferroptosis inhibitor (ferrostatin-1) in a clonogenic survival assay. Cells were plated at varying clonigenic densities after treatment with PTL or treatment with 500 nM Ferrostatin-1, 24 h before treatment with PTL **(A)** IC50 (18 µM), IC90 (54 µM), or **(B)** IC50 (13 µM) and IC90 (40 µM) (n= 6–12 clonogenic dishes plated from 3 separate treatment dishes). Errors represent ± 1 SEM. Significance determined by one-way ANOVA with Tukey’s post-analysis * *p* < 0.05.

Demonstration of elevation in lipid peroxidation in Figure 5 along with the decrease of GPx4 present within liver cancer cells (Figure 6) indicates ferroptosis as a possible mechanism for cell death. To confirm ferroptosis is occurring after exposure to PTL, we added ferrostatin-1 (an inhibitor of ferroptosis) to cells prior to treatment with PTL. The cell survival assay demonstrates a partial rescue after 6 h in both human (Figure 7A) and rat (Figure 7B) HCC cells. Figure 7 indicates that ferroptosis is a mechanism of cell death but not the only mechanism since cells were not fully rescued by ferroptosis inhibition.

### 3.7 PTL causes mitochondrial dysfunction

**FIGURE 8:**
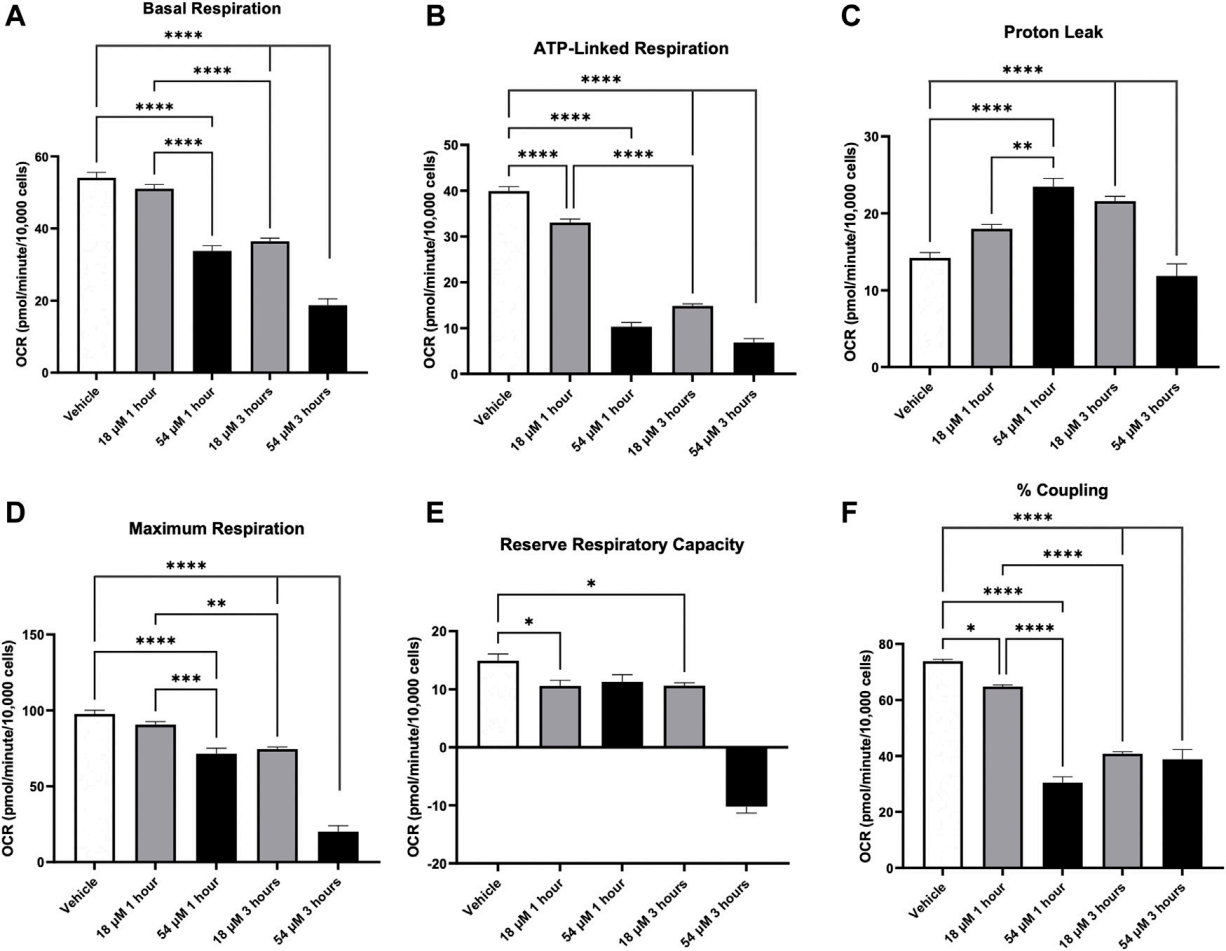
PTL resulted in mitochondrial dysfunction in HepG2 cells indicated by a decrease in basal respiration **(A)**, ATP-linked respiration **(B)**, elevation in proton leak **(C)**, decreases in both maximum respiration **(D)** and reserve respiratory capacity **(E)**, and decrease in % coupling **(F)**. HepG2 cells were treated with the IC50 (18 µM) or IC90 (54 µM) dose of PTL before injection of mitochondrial inhibitors or uncouplers to determine oxygen consumption rate (OCR) at 1 h and 3 h (n=3) Errors represent ± 1 SEM. Significance determined by one-way ANOVA with Tukey’s post-analysis * *p* < 0.05, ** *p* < 0.01, *** *p* < 0.001, **** *p* < 0.0001.

Since mitochondrial thiol oxidation was elevated after 24 h exposure to PTL (Figure 2C), we wanted to assess how this oxidation interferes with mitochondrial function and respiration. Using the Seahorse Flux analyzer, Figure 8 indicates that overall basal respiration (Figure 8A) was rapidly decreased after 1 h. Additionally, there was a decrease in mitochondrial coupling efficiency (Figure 8F) and ATP linked mitochondrial respiration (Figure 8B) indicating that PTL exposed mitochondria are no longer generating ATP efficiently. Moreover, the electron transport chain is leaking more protons (Figure 8C) further supporting the conclusion that mitochondria are functioning abnormally. Overall, PTL’s oxidation of mitochondrial thiols presents an early major disruption in mitochondrial activity which will lead to a vicious cycle of inefficient mitochondrial respiration, generation of more oxidative stress, and cell death.

## 4 Discussion

PTL is a natural extract with analgesic, anti-microbial, anti-inflammatory, and anti-cancer mechanisms that depend on a wide range of cellular signaling (Zhang et al., 2004). PTL’s action within the body does not appear to have one single mechanism but a variety of interactions with molecular targets such as nuclear factor kappa-light chain (NF-κB), activation of NADPH oxidases, thiol reactivity, and induction of apoptosis (Sobota et al., 2000; Pozarowski et al., 2003; Guzman et al., 2005; Sun et al., 2010). Previous studies have demonstrated that PTL’s anti-cancer activity within liver cancer cells is due to halted cell cycle progression at the G2/M phase by p53 activation and arrested cells in the G0-G1 through a decrease in cyclin D1 expression (Wen et al., 2002; Ralstin et al., 2006). Similarly, PTL induced apoptosis in HepG2 cells through decreased expression of anti-apoptotic Bcl-2 and elevated pro-apoptotic proteins that result in eventual activation of caspase-9 and caspase-3 (Sun et al., 2014). However, apoptosis is not the only cell death mechanism in response to PTL, as autophagy also occurs after PTL exposure within HepG2 cells, pancreatic cancer cells, osteosarcoma cells, and cervical cancer (Sun et al., 2014; D’anneo et al., 2013; Jeyamohan et al., 2016; Liu et al., 2017). Our present study confirmed that apoptosis was not the sole cell death mechanism occurring as ferroptosis played an important role in PTL’s cytotoxic effect in HCC cells.

Elevated oxidative stress can result in increased oxidative damage that creates a feed-forward cascade of generating ROS and leads to cell death (Berlett and Stadtman, 1997). Previous research attributed PTL’s activity to the rapid reaction of the lactone moiety with thiols (Herz, 1977). The present study found that PTL rapidly depleted intracellular thiols such as GSH by increasing oxidation and rapidly oxidizing mitochondrial thiols. These findings are comparable to what was found in colorectal cancer cells, where depletion of thiol groups on the mitochondrial membrane resulted in damage to the mitochondrial membrane’s integrity that caused a vicious cycle of oxidative stress-induced cancer cell death (Zhang et al., 2004). We found that in HCC cells, the elevation of mitochondrial thiol oxidation resulted in rapid deterioration of mitochondrial respiration and efficiency after 1 hour. Interestingly, the addition of a GSH precursor, NAC, 24 h prior was able to upregulate GSH levels such that treatment of HepG2 cells with IC90 dose which originally depleted GSH levels was able to compensate and respond to PTL by balancing GSH levels more effectively. Additionally, NAC was able to rescue HCC cells from PTL in the cytotoxicity assay, indicating that thiol oxidation is a significant mechanism of PTL toxicity within liver cancer cells. This study demonstrates that thiol oxidation from PTL occurs very quickly, leading to early mitochondrial dysfunction and oxidative stress.

Downstream effects from increased pools of oxidized GSH and rapid oxidation of intracellular thiols will leave cells vulnerable to other oxidizing reactions such as the oxidation of lipids (Ursini et al., 1989). Using the Image-IT lipid peroxidation sensor, we demonstrated an increase in lipid peroxidation after 6 h of exposure to PTL in HCC cells. This elevation of lipid peroxidation led us to consider the subsequent cellular mechanism of ferroptosis. Ferroptosis is a form of cell death that occurs from missing or insufficient activity of GPx4, resulting in increased lipid peroxidation (Lei et al., 2019). This is the first study to demonstrate ferroptosis as a mechanism of cell death in liver cancer cells. Our addition of ferrostatin-1 was able to partially rescue the cells indicating that ferroptosis is occurring; however, along with PTL’s other mechanisms of cell death, like apoptosis and autophagy, we were unable to fully rescue cells. Still, ferroptosis is a unique, irreversible cell death mechanism such that triggering ferroptosis will induce death in therapy-resistant cancer due to cancer cells relying on GPx4 for survival (Hangauer et al., 2017). We demonstrated that PTL reduces the protein levels of GPx4 within HepG2 cells, similar to what was found by Ding et al. (2021), that presented a combination of ferroptosis and apoptosis as a mechanism of cell death for triple-negative breast cancer cells. Loss of GPx4 may indicate why this elevation of lipid peroxidation and ferroptosis occurs at later time points.

Importantly, PTL has a pronounced differential effect on tumor cells compared to normal cells. A study by Wen et al. (2002) reported that PTL increases the total GSH in non-tumoral liver cells while causing a depletion of GSH in the cancerous liver cells. Furthermore, this elevation of GSH was also found in normal prostate cells for radioprotection (Sun et al., 2010; Xu et al., 2013). Likewise, we found that PTL was much less toxic to isolated primary murine hepatocytes than to HCC cell lines indicating that using an elevated cytotoxic dose of PTL for treatment of HCC will not be detrimental to normal liver parenchyma. However, a significant drawback of using PTL is its low bioavailability due to the predominantly lipophilic nature (Zhao et al., 2016). This unique characteristic of PTL may benefit patients with HCC, as the majority of patients are not eligible for curative systematic treatment and require unique options such as trans-arterial chemoembolization (TACE). TACE allows for the delivery of therapeutic treatment directly to the tumor with minimal side effects by avoiding systemic metabolism (de Baere et al., 2016; Stark et al., 2020). TACE can result in a 60 percent response rate sustained for 6 months or longer (Llovet and Bruix, 2003; Zhang et al., 2018). However, currently, TACE-treated tumors tend to re-vascularize, leading to increased recurrence rates (Park et al., 2013). The most common drug used in TACE is doxorubicin; nevertheless, approximately 50 percent of tumors treated with doxorubicin show no response, and only 30 percent show a complete response (Lammer et al., 2010). Thus, it is essential to consider alternative options to ensure that the treatment for patients with HCC is effective. Based on this study, we believe that PTL could be utilized as a therapeutic agent for TACE and plan to study further *in vivo* mechanisms and responses to PTL delivered locoregionally in a McA-RH777 orthotopic tumor model.

## Data Availability

The original contributions presented in the study are included in the article/Supplementary Material, further inquiries can be directed to the corresponding author.
