# Supplementary material for: Parthenolide induces rapid thiol oxidation that leads to ferroptosis in hepatocellular carcinoma cells
Source: Front Toxicol. 2022 Dec 14;4:936149. doi: 10.3389/ftox.2022.936149 (PMC9795200; doi:10.3389/ftox.2022.936149)
Supplement: Supplementary file 1 [file DataSheet1.PDF]

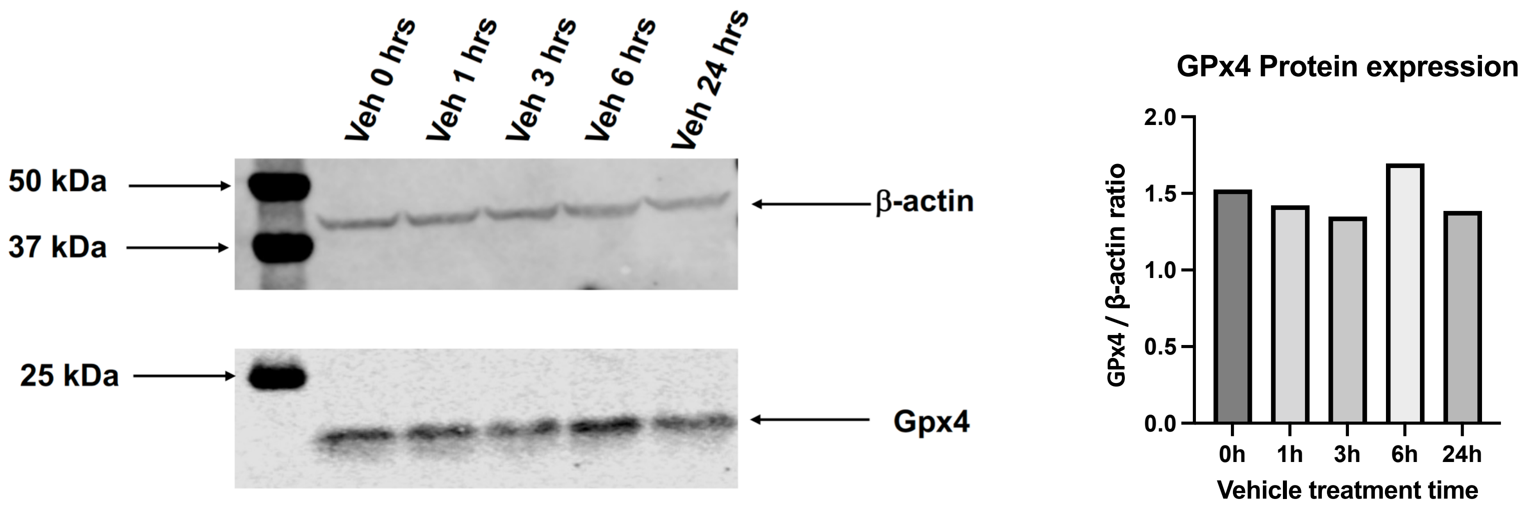

Figure S1. Protein expression of GPx4 was not altered when HepG2 cells were treated with 0.05% DMSO for 1, 3, 6 or 24 h compared to control (vehicle 0 h).

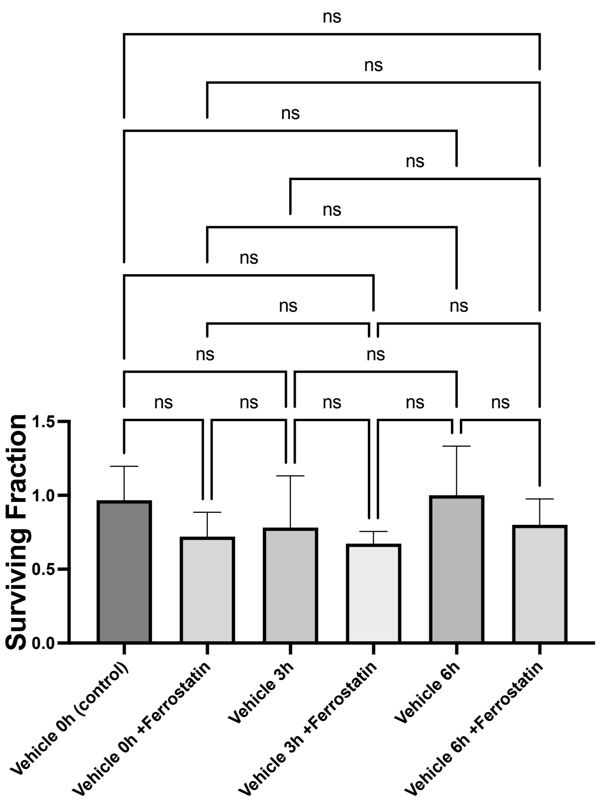

Figure S2. Treatment with DMSO vehicle (0.05%) in the presence and absence of ferrostatin (500 nM) did not change clonogenic survival of HepG2 cells at 3 or 6 h compared to control. Errors represent ± 1 SD. (n=3).

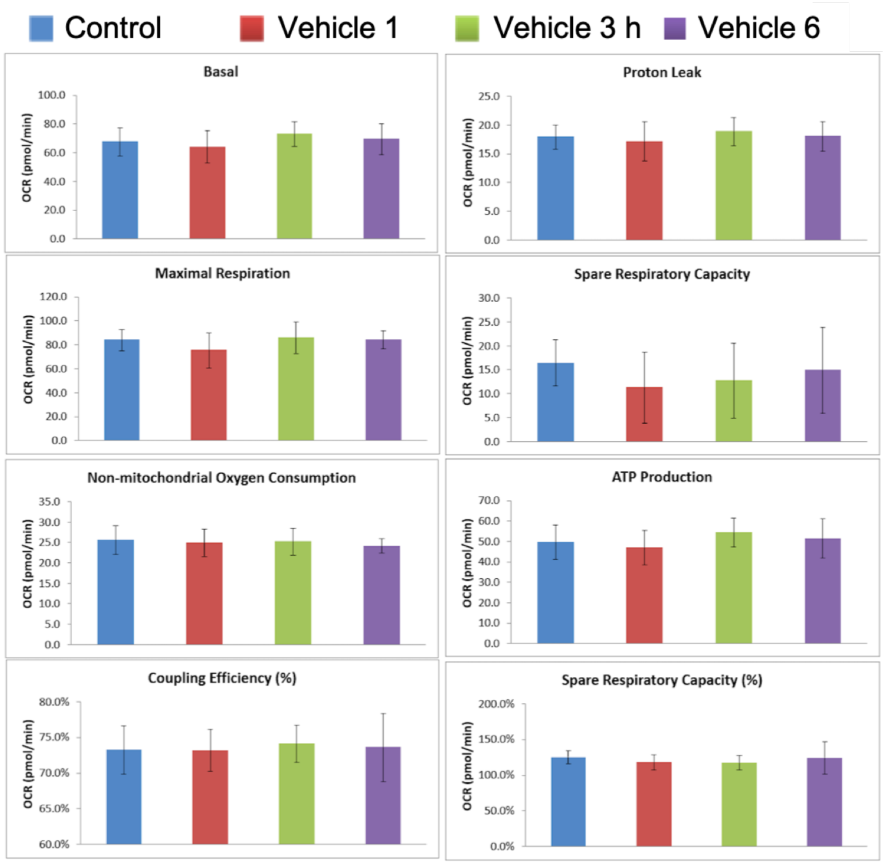

Figure S3. Treatment of HepG2 cells with DMSO vehicle (0.05%) did not alter any of the mitochondrial respiration and cellular bioenergetic parameters at 1, 3 or 6 h compared to control. Errors represent ± 1 SD. (n=8-12).
